# Erythropoietin reduces neuronal cell death and hyperalgesia induced by peripheral inflammatory pain in neonatal rats

**DOI:** 10.1186/1744-8069-7-51

**Published:** 2011-07-21

**Authors:** Osama Mohamad, Dongdong Chen, Lingling Zhang, Cane Hofmann, Ling Wei, Shan Ping Yu

**Affiliations:** 1 Department of Anesthesiology, Emory University School of Medicine, Atlanta, GA 30322; 2 Department of Neurology, Emory University School of Medicine, Atlanta, GA 30322; 3 Department of Pathology, Medical University of South Carolina, SC 29425

**Keywords:** pain, erythropoietin, neonates, inflammatory, cerebral blood flow, cell death

## Abstract

Painful stimuli during neonatal stage may affect brain development and contribute to abnormal behaviors in adulthood. Very few specific therapies are available for this developmental disorder. A better understanding of the mechanisms and consequences of painful stimuli during the neonatal period is essential for the development of effective therapies. In this study, we examined brain reactions in a neonatal rat model of peripheral inflammatory pain. We focused on the inflammatory insult-induced brain responses and delayed changes in behavior and pain sensation. Postnatal day 3 pups received formalin injections into the paws once a day for 3 days. The insult induced dysregulation of several inflammatory factors in the brain and caused selective neuronal cell death in the cortex, hippocampus and hypothalamus. On postnatal day 21, rats that received the inflammatory nociceptive insult exhibited increased local cerebral blood flow in the somatosensory cortex, hyperalgesia, and decreased exploratory behaviors. Based on these observations, we tested recombinant human erythropoietin (rhEPO) as a potential treatment to prevent the inflammatory pain-induced changes. rhEPO treatment (5,000 U/kg/day, i.p.), coupled to formalin injections, ameliorated neuronal cell death and normalized the inflammatory response. Rats that received formalin plus rhEPO exhibited normal levels of cerebral blood flow, pain sensitivity and exploratory behavior. Treatment with rhEPO also restored normal brain and body weights that were reduced in the formalin group. These data suggest that severe inflammatory pain has adverse effects on brain development and rhEPO may be a possible therapy for the prevention and treatment of this developmental disorder.

## Introduction

Clinical and basic studies have shown that early exposure to pain, particularly during periods of high brain plasticity and vulnerability in the neonates, alters normal neuronal connections and causes anatomic, electrophysiological and molecular changes that manifest as neurologic deficits in adolescence and adulthood [1,2]. Adults who were prematurely born are particularly more likely to suffer from altered states of pain sensitivity, learning and psychiatric disorders, hyperactivity and attention deficit disorders [3]. A potential mechanism for the behavioral alterations may be related to the observation that repetitive pain induces neuronal activation and widespread neuronal death in the brain [4]. The benefits of treating the neuronal cell loss in preventing the behavioral deficits, however, have not been studied.

Premature infants are babies born before completing 37 weeks of pregnancy. About 50,000 premature infants are born every year in the United States [5] reflecting a 20% increase in the last two decades [6]. Prematurity exposes infants to an environment that their bodies are not able to cope with yet. In the Neonatal Intensive Care Unit (NICU), premature infants undergo several tissue damaging procedures that induce extensive pain. For years, it was believed that infants do not feel pain as adults do. However, it has been recently shown that newborn infants do experience pain and, in fact, they may be even more sensitive than adults because they lack fully developed descending inhibitory tracts in the brainstem and spinal cord [7]. Zhuo and Gebhart showed that neonatal capsaicin treatment had significant effects on the bulbospinal systems important for the modulation of spinal nociceptive transmission. Specifically, capsaicin treatment of neonates leads to a reduction in inhibitory and an increase in facilitatory regulation on spinal nociception descending from the caudal brainstem [1]. In addition, sensory fibers relaying pain and mechanical information initially overlap during development making mechanical stimulation in neonates painful [8]. Despite the individual, social and economic burden of prematurity, relatively little effort has been invested to investigate the possibility that painful experiences during the neonatal period may have lasting impacts on brain development and behavioral complications in children and adults. Moreover, there are no FDA approved drugs specifically designed to ameliorate the consequences of prematurity or mitigate the long-term effects of the resulting developmental disorder. Some investigators suggested the use of the analgesic drug ketamine to reduce cell death in the brain and reverse the behavioral sequel of early repetitive pain [4]. Arguably, ketamine itself can cause neurodegeneration in immature cells of the developing brain especially at high dosages [9]. Several other pharmacological drugs have been tested for reducing the impact of pain in neonates. While topical anesthetics have failed clinical trials [10-12], the use of opioids is not favored because of its adverse effects on neuronal development [13]. Several other non-pharmacological interventions have been explored for the same purpose with variable efficacies such as nutritional support [14] and maternal holding and skin-to-skin care [15-17].

Erythropoietin is a glycoprotein produced by the kidney under conditions of systemic hypoxia to stimulate erythropoiesis [18]. Recombinant human erythropoietin (rhEPO) has been used for the treatment of anemia, myelodysplasia and some other illnesses [19,20]. Because of its multi-level protective effects and its safety profile, rhEPO has been tested in CNS disorders as well. For example, rhEPO showed neuroprotective effects after cerebral ischemia [21]. Administration of rhEPO after ischemic stroke reduces infarct volume [22], apoptotic cell death [23], and inflammation [24]. rhEPO enhances neurogenesis and angiogenesis and improves functional recovery following ischemic stroke [25]. rhEPO has been also found to be safe and efficacious in a preliminary clinical trial of human stroke patients [26]. Interestingly, rhEPO reduces pain after peripheral nerve injury [27] and in rheumatoid arthritis [28] most likely due to its anti-inflammatory action.

Given the prominent and proven therapeutic effects of rhEPO in the CNS, we hypothesized that the neuroprotective and anti-inflammatory actions of rhEPO could be used to reduce inflammatory pain-associated cell death in the brain and ameliorate the long-term behavioral consequences of repetitive painful stimuli during the early postnatal days.

## Materials and methods

### Chemicals

Formalin was purchased from VWR international (West Chester, PA). Recombinant human erythropoietin (rhEPO) was purchased from Amgen Biologicals (Thousand Oaks, CA).

### Animals and inflammatory pain model

The animals were kept under standardized temperature, humidity, and lighting conditions, with free access to water and food. Postnatal day 3 (P3) rat pups were subjected to 5% formalin (10 μl) subcutaneous (s.c.) injections into the four paws. rhEPO was administered 30 min before formalin injection in the EPO treated group. Some animals were injected once and sacrificed 4 or 12 hours later; while others were injected for three consecutive days and sacrificed on postnatal day 6 or 21 (P6 or P21). Pups were returned to their mothers immediately after each injection. The health condition of each pup was monitored each day for feeding behaviors and signs of feet swelling, skin infection, and pain development. Animals with skin infection, excessive swelling and pain were sacrificed without further testing. Mild paw swelling and skin redness recovered fully within 3 days after injection. After sacrifice, brain and dorsal root ganglia (DRG) tissues were collected for immunostaining and/or Western blot analysis. All studies were approved by the Institutional Animal Care and Use Committee (IACUC).

### Histochemical staining

Under isofluorane anesthesia, animals were transcardially perfused with 10% formalin. Brain coronal sections were sliced at 20 μm thickness using a cryostat vibratome (Ultapro 5000, St louis, MO, USA) and were mounted on gelatin coated slides. Fluoro-Jade C (FJC) staining [29], a valid and reliable stain for degenerating neurons, was conducted as reported earlier with some modifications. In brief, tissue sections were dried for 15 minutes. After three PBS washes, the sections were immersed in 0.8% NaOH in 80% ethanol followed by 70% ethanol and water washes, respectively. The sections were then immersed in 0.06% KMnO_4_, followed by 0.0002% FJC. The section were thereafter washed, dried and mounted for microscopy using the FITC channel on an upright Olympus fluorescence microscope.

### Terminal deoxynucleotidyl transferase Biotin-dUPT nick end (TUNEL) staining

TUNEL staining was performed using a commercial kit (DeadEnd™ Fluorometric TUNEL system, Promega, Madison, WI, USA) to label dying and dead cells in the brain. The instructions were followed as given. In brief, brain sections were placed in equilibration buffer and incubated with nucleotide mix and rTdT enzyme at 37°C for 1 hr. The reaction was stopped with 2 × SSC. Results were visualized using the FITC channel on an upright Olympus fluorescence microscope.

### Cell counting

Cell count was performed following the principles of design based stereology. Systematic random sampling was employed to ensure accurate and non-redundant cell counting [30]. Every section under analysis was at least a 100 μm away from the next. A total of 6 20-μm thick sections spanning the entire regions of interest were randomly selected for cell counting from each animal. Counting was performed on 6 non-overlapping randomly selected 20 × fields per section. Sections from different animals represent the same area in the anterior posterior direction.

### Western blot assays

Brain tissue samples were collected, proteins were extracted with lysis buffer, and protein concentration was measured with a BCA assay (Pierce, Rock Ford, IL, USA). 30 μg protein samples were electrophoresed on 6 to 15% gradient gels in the presence of sodium dodecyl sulfate-polyacrylamide in a Hoefer Mini-Gel system (Amersham Biosciences, Piscataway, NJ, USA) and transferred in the Hoefer Transfer Tank (Amersham Biosciences, Piscataway, NJ, USA) to a polyvinylidene difluoride membrane (BioRad, Hercules, CA, USA). Membranes were blocked in 7% milk (Tris-buffered saline containing 0.1% Tween-20, pH 7.6%, 7% milk) at room temperature for 1 h and incubated overnight at 4°C with rabbit polyclonal anti-caspase-3 (1:500, Cell Signaling, Boston, MA) and rabbit anti-apoptosis inducing factor AIF (1:1000, Cell Signaling, Boston, MA) antibodies. Mouse anti-actin antibody (1:5000, Sigma, St Louis, MO, USA) was used to detect β-actin, the protein loading control. The blots were washed in 0.5% Tris-buffered saline containing 0.1% Tween-20 and incubated with alkaline phosphatase conjugated anti-rabbit or anti-mouse (1:2000, Promega, Madison, WI, USA) antibodies for 2 hrs at room temperature. Finally, membranes were washed with Tris-buffered saline containing 0.1% Tween-20 followed by three washes with Tris-buffered saline. The signal was detected by the addition of bromo-chloro-indolyl phosphate/nitro blue tetrazolium (BCIP/NBT) solution (Sigma, St Louis, MO, USA), and quantified and analyzed by the imaging software Photoshop Professional (Adobes Photoshop CS 8.0, San Jose, CA, USA). This whole procedure was repeated three times with new samples collected from different animals.

### Quantitative real-time polymerase chain reaction (qRT-PCR)

Total RNA was extracted from the somatosensory cortex (4 and 12 hours after single injection and after 3 days' consecutive injections at P6 and P21) and dorsal root ganglia (12 hours after single injection) using the TRIzol reagent (Invitrogen Inc, Carlsbad, CA). Reverse transcription was performed with 2 μg total RNA using the High Capacity cDNA Reverse Transcription kit (Applied Biosystems, CA, USA). SYBR green qRT-PCR was used to assess the relative levels of our target genes using the Applied Biosystems StepOnePlus machine. The primers used were: TNF-α: F-primer: CCCCATTACTCTGACCCCTT; R-primer: TTGTTGGAAATTCTGAGCCC; Il-6: F-primer: GCCCTTCAGGAACAGCTATG; R-primer: CCGGACTTGTGAAGTAGGGA; Il-1β: F-primer: CATCTTTGAAGAAGAGCCCG; R-primer: AGCTTTCAGCTCACATGGGT; substance P (SP): F-primer: TGACCAAATCAAGGAGGCAAT; R-primer: GGGTCTTCGGGCGATTCT; Calcitonin gene related peptide (CGRP): F-primer: AAGTTCTCCCCTTTCCTGGTTGTCA; R-primer: TGGTGGGCACAAAGTTGTCCTTCAC;

Fold change was calculated by the delta (delta Ct) method using the 18S ribosomal RNA subunit amplification as the internal control. All experiments were repeated three times using distinct animals by two different investigators.

### Behavioral tests

To evaluate the behavioral changes in the experimental and control groups, we utilized the following behavioral tests.

#### Hot-Plate test

Pain sensitivity was measured on a hot plate at 55 +/- 1°C. Latency was measured as the time for the rat to jump, shake its limb or lick its paw with a maximum allowed time of 30 sec. Latency was measured as an average of three readings separated by at least 15 minutes.

#### Tail-flick test

The tail-flick test was performed using 55°C water as the nociceptive stimulus. The latency to remove the tail was measured three times separated by at least 15 minutes.

#### Defensive-withdrawal test

Animals were tested for exploratory behavior using the defensive withdrawal test for 10 min on postnatal day 21. During the test, the animals were placed in a dark chamber (10 × 15 × 10 cm high) placed along the wall of a large box (50 × 40 × 50 cm high). Animals were habituated to the open field 24 hours prior to behavioral testing by allowing them to explore the open field for 15 min without access to the defensive withdrawal chamber. The latency was measured as the time it takes the animal to place its four paws outside the small chamber. The experiment was performed by two separate individuals blinded to the identity of the groups.

#### Locomotor function tests

Motor function was assessed by the Rota-rod and open field tests. In the *Rota-rod test*, we measured the latency to fall off the rotating rod with a maximum cut-off time of 6 min. Averages of three trials, separated by at least 15 min, were calculated. In the *open-field test*, rats were allowed to freely move in an open field container (50 cm × 40 cm × 50 cm high) during the dark cycle. The container was divided into 16 equal squares and the number of line crossings by each animal was calculated for 5 min.

### Cerebral blood flow measurement

Cerebral blood flow was measured using laser-Doppler flowmetry (PeriFlux System 5000 - PF5010 LDPM unit, Perimed, Stockholm, Sweden). The tip of a flexible 0.5 fiber optic probe was pointed over the somatosensory cortex. Measurements were taken before and after the last injection of formalin on postnatal day 5 during the third injection session. The final value is the ratio of blood flow after to before injections and it is an average of five consecutive readings.

### Statistical analysis

All analyses were performed using GraphPad Prism 4.0 statistical software (GraphPad Software, Inc., La Jolla, CA). Multiple comparisons were performed by one- or two-way analysis of variance (ANOVA) followed by Bonferroni's post hoc analysis. Single comparisons were performed using Student's t-test. Changes were considered significant if the p-value was less than 0.05. Mean values were reported with the standard error of the mean (SEM).

## Results

### Formalin-induced peripheral inflammatory pain model in neonatal rats

The subcutaneous formalin administration into the paws of rodents is a well established nociceptive insult used in peripheral inflammatory pain models [31,32]. P3 neonatal rats were randomized to four groups: saline injection controls, rhEPO injection controls, formalin injection group and formalin coupled to intraperitoneal (i.p.) injection of rhEPO (5,000 U/kg). It is estimated that the rat brain at postnatal days 1-10 is equivalent to a third trimester human brain and that rat neurodevelopment at postnatal day 7 is equivalent to that of the human brain at birth [33,34]. The postnatal day 3 was selected to mimic premature birth in humans. rhEPO was administered 30 min before each formalin injection in the formalin plus EPO treatment group. Formalin (5%, 10 μl) was subcutaneously injected into the four paws, starting on P3. Animals sacrificed at 4 and 12 hours received only one treatment with saline control, rhEPO alone, formalin alone or formalin plus rhEPO, respectively. Animals sacrificed at P6 (72 hrs) and P21 received injections from P3 to P5 once a day in each paw. Rat pups receiving formalin injections showed skin redness and moderate swelling at injection sites. These changes fully reversed in 3 days. We regard the employed formalin model as a severe and subacute inflammatory pain model, suitable for generating noticeable short- and long-term alterations.

**Figure 1:**
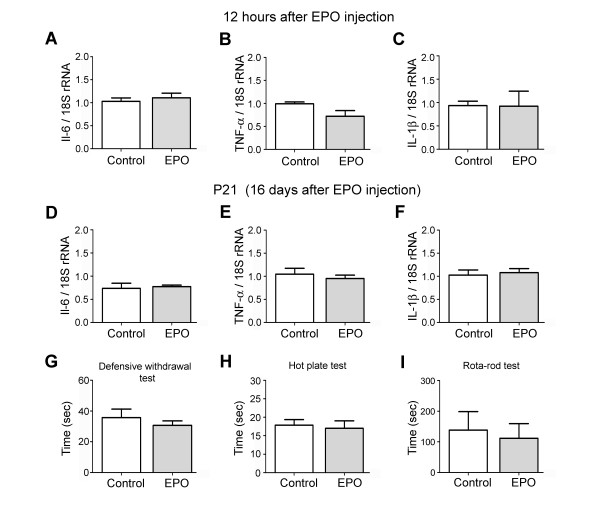
**Effect of rhEPO on cytokine expression and behavioral activities**. Control tests were performed to evaluate whether rhEPO injection affect inflammatory cytokine expression in neonatal rats or behavior in juvenile rats. **A - C**: The mRNA levels of IL-6, TNF-α and IL-1β in the somatosensory cortex 12 hrs after a single injection of rhEPO (5000 U/kg) in P3 neonatal rats. **D - F**: The mRNA levels of IL-6, TNF-α and IL-1β in the somatosensory cortex on postnatal day 21 after 3 injection sessions of rhEPO (5000 U/kg) in P3-P5 neonatal rats. **G - I**: rhEPO did not influence the behavior of juvenile rats that were injected three times with rhEPO at P3, P4 and P5. Saline injected controls and rhEPO injected animals performed similarly in the defensive-withdrawal (G), hot-plate (H) and the Rota-rod (I) tests (n = 4 for each group).

As a control study for identifying the effect of rhEPO on cytokine expression and animal behavior, we injected rhEPO alone in P3 neonatal rats and inspected cytokine expression 12 hrs later, and cytokine expression and behavior at P21. Injection of rhEPO into P3 neonatal rats did not affect IL-6, TNF-α and IL-1β transcription levels 12 hrs later (Figure 1A to 1C). Moreover, rhEPO, injected three times in neonatal rats at P3, P4 and P5, did not affect transcription of inflammatory cytokines (IL-6, TNF-α and IL-1β) examined at P21 (Figure 1D to 1F). In addition, the behavior of P21 juvenile rats (injected with rhEPO at P3, P4 and P5) was not affected in the defensive-withdrawal, hot-plate or Rota-rod tests (Figure 1G to 1I).

### rhEPO treatment reduced inflammatory pain-induced cell death in the neonatal brain

**Figure 2:**
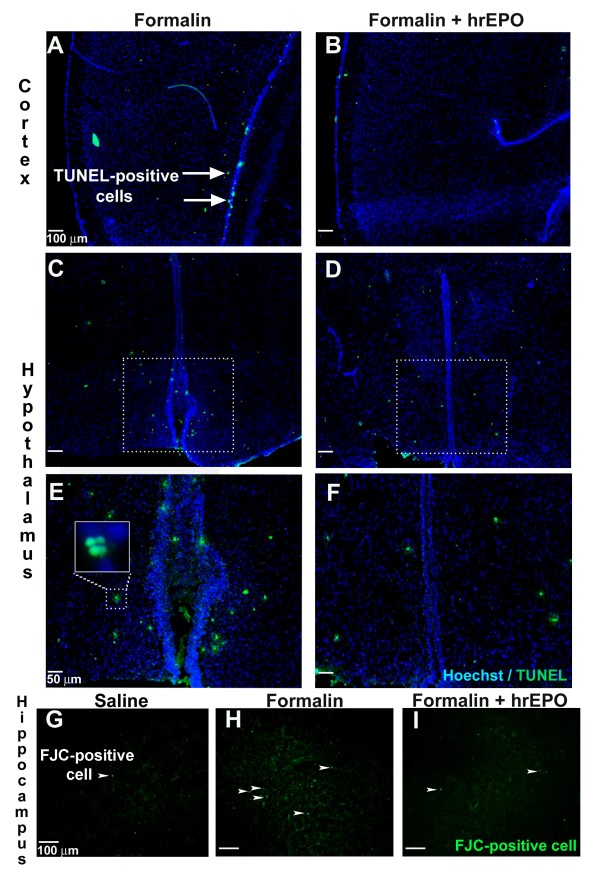
**Effect of rhEPO on inflammatory pain-induced cell death**. rhEPO reduced cell death in the cortex, hypothalamus and hippocampus after 3 formalin injection sessions (72 hrs). **A **- **F**: Immunofluorescent images of TUNEL-positive cells (arrow) in the cortex (A and B) and the hypothalamus (C and D). E and F are magnified selected areas in C and D, respectively. The inset in E is a further enlarged (40×) image of the selected area (Blue: Hoechst, green: TUNEL). **G - I**: Immunofluorescent images of fluoro-Jade C positive neurons (arrowheads) in the hippocampus after 3 repetitive saline (G), formalin (H) or formalin plus rhEPO injection (I) sessions (green: FJC-positive cell). Bar = 100 μm for A to D, 50 μm for E and F, and 100 μm for G to I.

**Figure 3:**
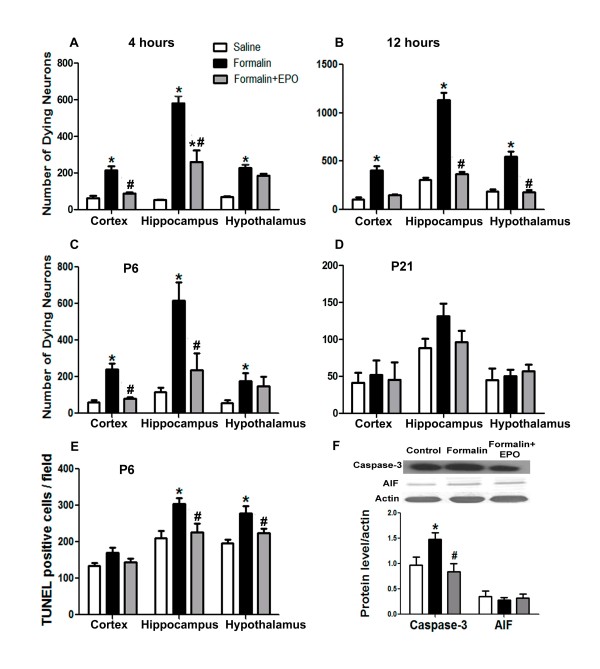
**Neuroprotective effect of rhEPO**. rhEPO reduced cell death after repetitive formalin injections in the cortex, hypothalamus and hippocampus after repetitive formalin injections. **A **- **D**: Quantifications of the number of fluoro-Jade C positive neurons in the cortex, hippocampus and hypothalamus 4 (A) and 12 hrs (B) after a single injection or after 3 injections at P6 (C) and P21 (D). **E**: Quantification of TUNEL-positive cells in the cortex, hippocampus and hypothalamus after 3 injection sessions (n = 6 in each group). *. p < 0.05 compared to saline group; #. p < 0.05 compared to formalin group. **F**: Western blot analysis of somatosensory cortex proteins from saline, formalin or formalin plus rhEPO groups. Cleaved caspase-3 was measured in total lysates and Apoptosis Inducing Factor (AIF) was inspected in the cytosolic extracts 72 hrs after 3 injections sessions. β-actin was used as the loading control. N = 4 for caspase-3 and n = 6 for AIF. *. p < 0.05 compared to saline group; #. p < 0.05 compared to formalin group.

TUNEL staining was used to investigate the extent of cell death in the brain. TUNEL identifies fragmented DNA in all types of dying cells [35,36]. In addition, we used Fluoro-Jade C (FJC) to specifically identify dying neurons [29]. The analysis of data from TUNEL staining after 3 sessions of formalin injection (72 hrs) revealed that the peripheral inflammatory nociceptive insult caused widespread cell death in the cortex and hypothalamus (Figure 2A, C and 2E), whereas rhEPO co-treatment effectively reduced cell death in these areas (Figure 2B, D, and 2F). Counting of TUNEL-positive cells in the cortex, hippocampus and hypothalamus showed a significant effect of rhEPO in reducing cell death after repeated formalin injections (Figure 3E). In the hippocampus and hypothalamus, rhEPO treatment showed significant protection. TUNEL positive cells were reduced from 303 ± 44/field in the formalin alone rats to 227 ± 59/field, and from 278 ± 50/field to 224 ± 32/field in these two regions, respectively (n = 6 and p < 0.05 in both analyses). The number of TUNEL-positive cells in the cortex of rhEPO-treated rats was 145 ± 24 as compared to 170 ± 37/field in the formalin alone group (however, p > 0.05, n = 6 rats in each group). The more specific neuronal stain FJC further verified that treatment with rhEPO dramatically reduced neuronal cell death in the cortex, hippocampus (Figure 2G to 2I) and hypothalamus after a single (4 and 12 hrs) or 3 formalin injections (72 hrs) (Figure 3A to 3C). For example, 12 hrs after a single injection, rhEPO reduced FJC-positive cells from 408 ± 41/field to 148 ± 12/field in the cortex, from 1136 ± 71/field to 368 ± 23/field in the hippocampus, and from 548 ± 50/field to 182 ± 21/field in the hypothalamus (p < 0.05 vs. saline control in all three comparisons; n = 6 in each group) (Figure 3B). At age P21, the number of dying neurons was similar in the control, formalin injected and EPO treated groups and they were significantly lower than the earlier time points (Figure 3D).

### rhEPO reduced formalin-induced apoptotic cell death in the neonatal brain

Immature neurons are more vulnerable to apoptotic insults [37]. We tested the hypothesis that formalin injection triggers the activation of apoptotic mechanisms in the neonatal brain. To check for this possibility, we performed Western blot analysis on somatosensory cortical tissues collected after 3 formalin injections in 3 days and probed for the levels of caspase-3 and apoptosis-inducing factor (AIF). Formalin injections led to a significant rise in the levels of cleaved caspase-3 compared to saline group (1.48 ± 0.13 vs. 0.97 ± 0.17 folds; p < 0.05), whereas rhEPO co-treatment strongly prevented caspase-3 activation (0.84 ± 0.16 vs. 1.48 ± 0.13 folds; p < 0.05) (Figure 3F). The formalin insult did not change the cytosolic levels of AIF, which represents the caspase-independent apoptosis [38].

### rhEPO modified the inflammatory response by regulating cytokine levels

**Figure 4:**
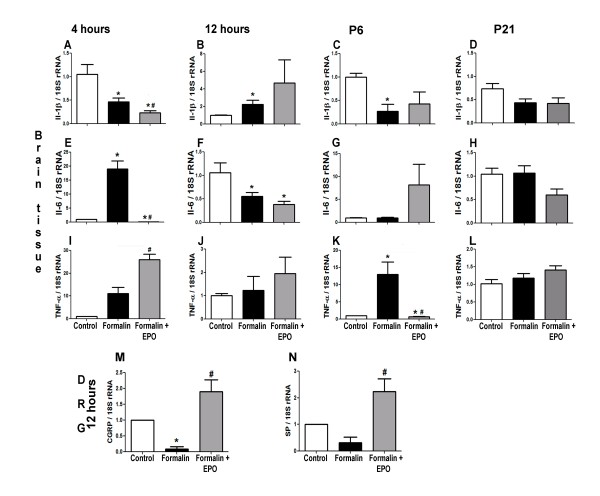
**Effects of rhEPO on expression of inflammatory factors**. Real time PCR measurements of inflammatory factors in somatosensory cortex or DRG from control and experimental rats. **A **- **D**: The mRNA levels of IL-1β in the somatosensory cortex 4 and 12 hrs after a single injection or after 3 injection sessions (72 hrs and P21) of saline, formalin or formalin plus rhEPO groups. **E - H**: The mRNA levels of IL-6 in the somatosensory cortex 4 and 12 hrs after a single injection or after 3 injection sessions (72 hrs and P21) of saline, formalin or formalin plus rhEPO groups. **I - L**: TNF-α mRNA levels in the somatosensory cortex 4 and 12 hrs after a single injection or after 3 injection sessions (72 hrs and P21). **M **and **N**: The mRNA levels of CGRP (J) and substance P (SP) in DRG neurons 12 hrs after a single injection. All results are expressed as fold changes compared to the control. 18S rRNA was used as an internal control. n = 6 in each group; *. p < 0.05 compared to saline group; #. p < 0.05 compared to formalin group.

To further characterize the mechanisms by which formalin caused widespread cell death in the brain, we aimed to identify the transcriptional levels of some major inflammatory cytokines involved in the pathogenesis of inflammatory pain. To measure transcription, we used real-time PCR to follow mRNA production of IL-6, IL-1β, and TNF-α in the somatosensory cortex and substance P (SP) and calcitonin-gene-related peptide (CGRP) in the dorsal root ganglia (DRG) corresponding to the upper and lower limbs. In the somatosensory cortex, formalin injection significantly increased Il-6 at the 4 hrs time point (19.0 ± 3.0 folds increase; p < 0.05) (Figure 4E). TNF-α level was significantly enhanced at the 4 hr (11.1 ± 2.7 folds) and 72 hr (13.0 ± 3.5 folds) time points (p < 0.05 in both comparisons) (Figure 4I and 4K). IL-1β level increased 2.3 ± 0.5 folds (p < 0.05 compared to controls) (Figure 4B) at the 12 hrs time point. On the other hand, formalin injection reduced IL-1β levels at 4 and 72 hrs after injection (Figure 4A and 4C). rhEPO treatment effectively prevented the increases in IL-6 and TNF-α levels at 4 and 72 hrs, respectively (Figure 4E and 4K). However, rhEPO did not reduce the levels of TNF-α 4 hrs after formalin injection. TNF-α transcription level was still high at this early time point (Figure 4I). In addition, there was no significant difference in the transcriptional levels of IL-1β, IL-6, or TNF-α when measured at age P21 after 3 injection sessions (Figure 4D, H and 4L). Moreover, we detected a strong trend of decreased SP level (0.31 ± 0.20 folds vs. the control; but p > 0.05) and a decreased CGRP level (0.09 ± 0.08 folds vs. the control; p < 0.05) 12 hrs after a single formalin injection. Treatment with rhEPO significantly increased the mRNA expression of SP (2.2 ± 0.2 folds vs. formalin alone; p < 0.05) and CGRP (1.9 ± 0.4 folds vs. formalin alone; p < 0.05) (Figure 4M and 4N).

### rhEPO treatment prevented abnormal pain sensitivities

**Figure 5:**
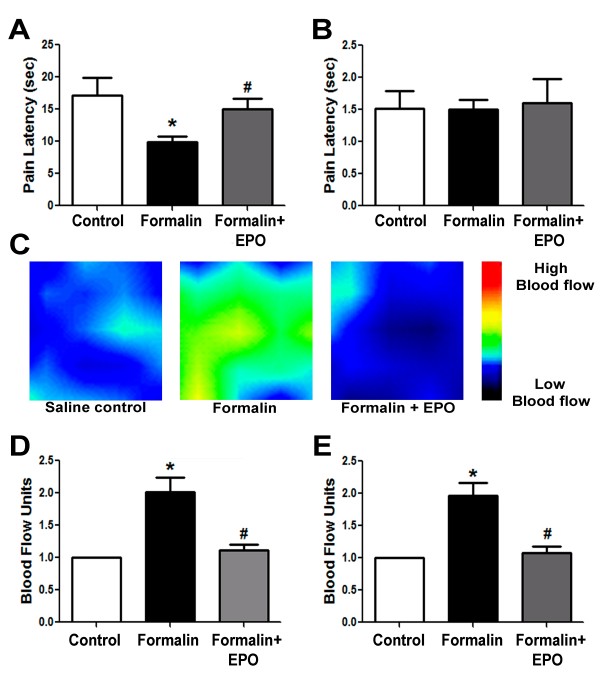
**Effect of rhEPO on pain sensitivity and cerebral blood flow of formalin-injected rats**. Rats were subjected to pain sensitivity hot plate and tail flick tests and cerebral blood flow was measured using a laser Doppler scanner. **A **and **B**: Measurements of pain latencies on hot-plate (A) and tail-flick (B) tests in 21 day old juvenile rats after different treatments during infancy (P3 to P5). rhEPO restores normal pain sensitivity after formalin injections. **C**: Representative laser Doppler graphs of blood flow levels in the right somatosensory cortex of P5 rats after the third injection of formalin or formalin plus rhEPO. The calibration bar on the right shows relative levels of flow. **D **and **E**: The ratio of blood flow after/before injections from experiments in C, in the right (D) and left (E) hemispheres. The values are averaged from five consecutive readings. N = 7 in pain tests and n = 6 in blood flow measurements; *. p < 0.05 compared to saline group; #. p < 0.05 compared to formalin group.

A major complication of early exposure to painful experiences is altered pain sensation in adult life [39,3]. This fact prompted us to test pain sensitivities in 21-day old juvenile rats after they have received three injection sessions of saline, formalin alone or formalin plus rhEPO in infancy (P3 to P5). We noticed that rats which received repetitive formalin injections had reduced pain thresholds in the hot-plate test (9.9 ± 0.9 sec vs. 17.1 ± 2.8 sec; p < 0.05). Interestingly, there was no difference in their pain thresholds in the tail-flick test (1.5 ± 0.2 sec vs. 1.5 ± 0.3 sec; p > 0.05) (Figure 5A and 5B), suggesting that the peripheral inflammatory stimuli selectively affect supra-spinal but not spinal pain pathways. Treatment with rhEPO prevented the hyperalgesia in the hot-plate test (Figure 5A), which is consistent with the rhEPO effect in L5 spinal nerve transection neuropathic pain model [40].

### Effect of rhEPO on increases in cerebral blood flow induced by inflammatory pain

To check if the altered pain sensitivity is associated with increased brain activity during the painful stimuli, we measured local cerebral blood flow (LCBF) in the brain 5 min before and immediately after formalin injection on the third and last day of injections in animals that have received three injection sessions (postnatal day 5). Formalin injection significantly increased blood flow in the right somatosensory cortex; whereas treatment with rhEPO restored normal flow (Figure 5C and 5D). Same results were obtained from the left hemispheres (Figure 5E). These data suggested that peripheral inflammatory pain causes a state of hypersensitivity/hyperactivity in the brain and rhEPO was able to restore normal brain activity levels upon the painful stimuli.

### rhEPO treatment improved exploratory behavior on defensive-withdrawal test

**Figure 6:**
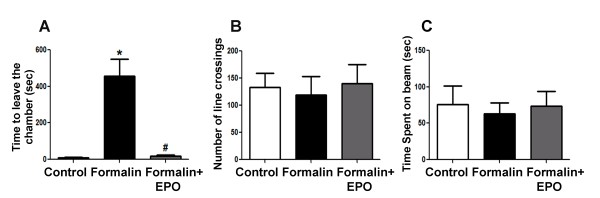
**Effect of rhEPO on abnormal exploratory behaviors induced by inflammatory pain**. Exploratory behaviors and motor functions were tested in juvenile rats that were exposed to control or three formalin injections with and without rhEPO in infancy. **A**: Quantification of the latency to leave the small chamber in defensive-withdrawal test in 21 day old rats. Rats in the formalin group hesitated to leave the chamber while rhEPO co-injection prevented this abnormal behavior. **B**: Rats were placed in an open field and their locomotion was monitored for 5 min. The numbers of lines rats crossed were counted and there was no difference between groups, suggesting unaffected motor function. **C**: The time rats spent on the rotating beam in Rota-rod test, showing no difference between groups. N = 7 in the defensive-withdrawal test and n = 5 in the Rota-rod and open-field tests; *. p < 0.05 compared to saline group; #. p < 0.05 compared to formalin group.

Behavioral disturbances and psychiatric disorders are major complications that stem from early painful experiences. Defensive-withdrawal test evaluates exploratory behavior in rats. After 3 sessions of formalin injections in the neonatal period, we tested exploratory behavior in the juvenile 21-day old rats. Formalin injections increased the time spent in the dark chamber (456 ± 95 sec vs. 10 ± 3 sec in control rats; p < 0.05). rhEPO treatment corrected this abnormal behavior back to the normal level (17 ± 8 sec; p > 0.05 compared with controls) (Figure 6A). To rule out the possibility that the formalin insult impaired locomotor functions, we tested locomotion using the Rota-rod and open field tests in the P21 rats. There were no statistically significant differences on either test among the three groups (Figure 6B and 6C).

### rhEPO treatment helped retain brain and body weights

**Figure 7:**
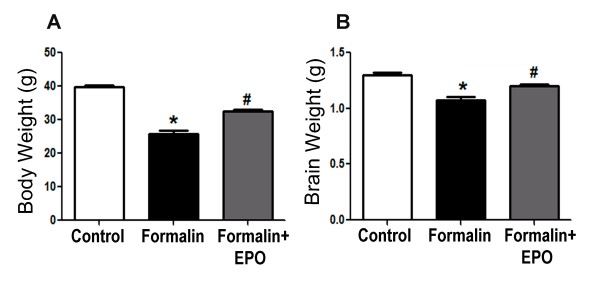
**Effect of rhEPO on body and brain weight loss induced by inflammatory pain**. Rats that received formalin injections during infancy underwent body and brain weight loss when growing up to juvenile age. **A **and **B**: Measurements of body and brain weights respectively in 21-day old rats subjected to 3 injections of saline, formalin or formalin plus rhEPO in infancy. Co-injecting rhEPO improved brain and body weights in these developing animals. N = 8 in each group; *. p < 0.05 compared to saline group; #. p < 0.05 compared to formalin group.

In the long-term experiments, we tested the effect of formalin injections on brain and body weights in the P21 rats of the three experimental groups. Rats that received formalin injections during their neonatal period showed significantly less body (25.8 ± 1.0 g vs. 39.7 ± 0.6 g; p < 0.05) and brain (1.1 ± 0.02 g vs. 1.3 ± 0.03 g, p < 0.05) weights compared to the control group. These decreases in body and brain weights were not seen in rats that also received rhEPO. Their body and brain weights remained within the normal ranges (Figure 7A and 7B).

## Discussion

The present investigation reveals that severe inflammatory nociceptive insults occurring in neonatal rats cause brain cell death and have significant adverse impacts on brain development and behavioral activities in adolescence. Specifically, rats that suffer painful inflammatory nociception in infancy developed hyperalgesia and abnormal stress behaviors. We show for the first time that rhEPO is an effective drug that antagonizes the inflammatory response and brain damage caused by inflammatory pain. rhEPO treatment prevents the development of hyperalgesia and corrects the stressful behavior chronically developed in these young animals. Moreover, treatment with rhEPO maintains normal brain and body weights that were reduced in animals suffering repetitive inflammatory stimuli.

Contrary to what was previously believed neonates can feel pain and, in many cases, pain is more intense than in adults due to the underdeveloped inhibitory mechanisms in the brain and spinal cord [1,7,8]. In clinical cases, painful stimuli and inflammatory pain occur in human infants. For example, prematurely born infants in the intensive care unit are subjected to painful procedures including, but are not limited to, heel sticks, endotracheal intubations, respiratory and gastric suctioning and catheter insertions [41]. It is noticed that these infants may develop behavioral and emotional problems in childhood, altered pain responses, depression and other psychiatric disturbances in adulthood [2]. The cellular, molecular and pathological mechanisms of this disorder are not well understood. Previous studies showed that full term neonates exposed to painful stimuli at birth develop acute adverse effects that last only for hours and are not chronic like those seen in premature babies [42,43]. It is likely that the data from the present investigation are more relevant to immature infants subjected to severe and repetitive inflammatory pain.

Formalin s.c. injection is used as a model for inflammatory pain. Formalin is usually applied as a fixative in immuno-histochemical staining. It fixes tissues by reversibly cross-linking primary amino groups in proteins with nearby nitrogen atoms. When injected subcutaneously, formalin causes extensive firing of primary afferent nerves such as the C-fibers and widespread signaling activities in the brain and spinal cord [44] resulting in a state of repetitive inflammatory pain in animals [31]. Formalin causes extensive neuronal activity that lead to glutamate excitotoxicity and increased neuronal death in the brain [45]. In line with these pathophysiological events, we observed high levels of TUNEL and FJC staining in the cortex, hippocampus and the hypothalamus after formalin injection. The cortical regions are the primary somatosensory and associative sensory areas, which receive input from the primary afferents through the thalamus. The hippocampus, on the other hand, is part of the limbic system and it extensively receives nociceptive input and mediates a variety of functions including emotions and behaviors, whereas the hypothalamus, part of the hypothalamic-pituitary-adrenal axis, mediates a variety of metabolic, neuroendocrine and autonomic activities in response to physiologic and pathologic stresses. The roles of these areas in mediating somatosensory and behavioral functions and excitotoxicity explain, at least partly, the consequences of the high cell death levels detected after formalin injection. A recent study showed that formalin stimulation in neonatal rats causes long-term behavioral changes, altering exploratory behavior and anxiety levels in adulthood in a gender (female) specific manner [46]. In that study, a relatively minor insult of single paw formalin injection was tested. It will be interesting to see if gender plays a role in the severe inflammatory pain model.

Previous work focused on the use of anesthetics and a few other non-pharmacological interventions to reduce pain and to prevent the emergence of long-term complications of early exposure to painful stimuli. However, given the controversial role and adverse effects of various anesthetics on the immature brain, for instance induction of neuronal apoptosis, these treatments have raised increasing concerns. The present investigation is the first attempt to utilize a neuroprotective agent, rhEPO, to prevent the long term complications of early painful experiences in neonates. rhEPO has been studied for its safety and efficacy in neonatal brain injury and for stimulating erythropoiesis in human infants. For example, extremely low birth weight infants who were given rhEPO at birth to stimulate erythropoiesis showed better mental capacities and improved developmental assessment at the age of 10 [47-49] although other reports failed to show neurodevelopmental benefits with rhEPO treatment [50]. It is important to mention, however, that the neuroprotective effects of rhEPO are achieved at doses higher than those required for its erythropoietic actions. Nonetheless, human infants did not develop any of the complications associated with rhEPO seen in adult patients like hypertension, clotting, polycythemia, and tumor angiogenesis [51,52]. Moreover, the erythropoietic actions of EPO are beneficial in preterm infants who are mostly anemic. We have also shown that rhEPO injections do not affect cytokine expression in control animals and that neonatal rhEPO treatment does not modify behaviors in adolescence. rhEPO was tested in this investigation as a preventative and protective treatment for neonates subjected to severe painful stimuli that cause adverse impacts on CNS cells and brain development. As the injury is predicted to occur and as rhEPO is a relatively safe drug, we believe it is feasible to use rhEPO to co-treat or pre-treat these neonates. Future studies may be performed to evaluate the therapeutic benefits of delayed treatment of rhEPO in older animals.

Subcutaneous formalin injections also cause non-specific tissue damage and activate other afferents that are not related to pain processing [53]. We cannot exclude that rhEPO may show a local anti-inflammatory effect at the site of formalin injections. We can confirm, however, that, based on the results of locomotion tests, the behavioral alterations in the formalin injected rats were not due to injured paws and impaired locomotor abilities. We have also demonstrated that rhEPO helps to restore brain and body weights compared to the formalin group. This is a likely indication of complex interacting factors including cell death, cell proliferation/regeneration and feeding behavior that is linked to the limbic/hypothalamic interaction.

EPO receptor (EPOR) is abundantly expressed in brain capillaries, and EPO can cross the blood brain barrier via endocytosis [21]. In neonatal rat brains, rhEPO reaches significant levels at 4 hours but peaks later at 10 hours after i.p. injections [54]. This explains the protective effect of rhEPO in reducing TUNEL and FJC staining in several brain regions. It is worth to mention that, when investigated in P21 juvenile rats, we could not identify any difference in the number of dying neurons among the three groups. This observation agrees with the idea that cell death that occurred during early brain development is critical. rhEPO has anti-apoptotic functions as shown in the reduction of cleaved caspase-3 in the present study. We additionally revealed that the inflammatory pain-triggered neuronal apoptosis is caspase-dependent but not AIF-dependent, since this caspase-independent apoptotic factor was not increased by formalin injections. EPO and EPOR are expressed in both neurons and glial cells in the central nervous system [55]. The binding of EPO to its receptor initiates a cascade that activates the ERK-1/-2, Akt and JNK-1/-2 signaling pathways [56]. These pathways eventually activate factors that inhibit apoptosis, decrease inflammation, increase neurogenesis and angiogenesis. EPO and EPOR also induce neurogenesis after stroke [57], early during embryonic development [58] and in vitro via Jak2/Stat3 and PI3K/AKT pathway activation [59]. We suggest that the therapeutic benefits of rhEPO treatment may be partly attributed to an increase in brain neurogenesis as well.

Several reports in the literature attribute an anti-inflammatory action to rhEPO in stroke and endotoxin-mediated inflammation [24,60]. Both TNF-α and IL-1β are pro-inflammatory [61] whereas IL-6 can either be pro- or anti-inflammatory [62]. In the present investigation, rhEPO reduced IL-1β, IL-6 and TNF-α at many time points whereas it increased TNF-α at the 4 hrs time point. It is worth to note that IL-6 significantly dropped in the formalin group while it increased in the rhEPO treated group along the 3 time points (from 4 to 72 hrs). TNF-α, on the other hand, significantly increased in the formalin group while it dropped in the rhEPO treated group in the same time span. In addition, it is well established that levels of SP and CGRP drop in the DRG in models of chronic pain [63] and in the spinal cord in a neonatal pain model [1]. We demonstrate, similar to other reports [64], that rhEPO can reverse signs of central sensitization (restoring normal levels of SP and CGRP). CGRP expression is regulated by an upstream MAPK-responsive enhancer element. Since EPO activates the MAPK signaling cascade, the increase in CGRP expression can be attributed to EPO-induced activation of this upstream MAPK-responsive enhancer [65]. Only mRNA levels were measured in this investigation, whether the protein levels of these factors are similarly changed remains to be confirmed.

A recent report shows that rhEPO enhances cognitive function of memory and learning [66]. This is in line with our observations in current investigation and, collectively, it is suggested that rhEPO and EPOR may be explored as targets in a preventive therapy for premature neonates subjected to repeated painful procedures.

## Competing interests

The authors declare that they have no competing interests.

## Authors' contributions

OM participated in the experimental design, carried out molecular genetic examination using Western blotting and qRT-PCR and performed behavioral experiments. He also contributed heavily in data analysis and manuscript writing. DC contributed to formalin injections, behavioral tests and data collection, LZ carried out formalin injections, immunohistochemistry, qRT-PCR, and behavioral tests, CH participated in immunohistochemistry and behavioral tests, LW contributed to experimental design and data analysis, SPY helped in concept development, data analysis, and manuscript writing.

All authors have read and approved the final manuscript.
